# Growth and Comprehensive Quality Index of Tomato under Rain Shelters in Response to Different Irrigation and Drainage Treatments

**DOI:** 10.1155/2014/457937

**Published:** 2014-06-25

**Authors:** Guang-cheng Shao, Ming-hui Wang, Na Liu, Min Yuan, Prem Kumar, Dong-Li She

**Affiliations:** ^1^College of Water Conservancy and Hydropower Engineering, Hohai University, Nanjing 210098, China; ^2^Key Laboratory of Efficient Irrigation-Drainage and Agricultural Soil-Water Environment in Southern China, Ministry of Education, Nanjing 210098, China; ^3^School of Economics and Management, Nanjing University of Information Science & Technology, Nanjing 210044, China; ^4^Indian River Research & Education Center, University of Florida, Fort Pierce, FL 34945, USA

## Abstract

The effects of two levels of irrigation water (100%, 60%) and buried underground pipe depths (0.8 m, 0.6 m) under rain shelters' conditions on yield and some quality parameters of tomato were investigated. A fully randomized factorial experiment was conducted between April and August in 2011 and 2012 at Hohai University. It was found that drainage treatments enhanced biomass production, whereas soil desiccation led to biomass reduction. At 60 cm buried underground pipe depths, the drought treatments increased the mean root weight and root-shoot ratio by 14% and 39%, respectively. The main effects of drainage treatments on the fruit quality were increases in total soluble solids (TSS), soluble sugar (SS), and vitamin C (V_C_) compared to the control. In addition, drainage treatments increased the average yield by 13% and 9%, respectively, in both years. The drought treatments did not significantly alter fruit yield, although mean single fruit weight was slightly reduced. Instead, these treatments tend to have great potential to improve fruit quality (TSS, SS, and V_C_) to variable extents. In both years, the drought treatment at 60 cm buried underground pipe depths proved to possess the highest comprehensive quality index based on Principal Component Analysis.

## 1. Introduction

Heavy rain, high temperature, and high relative humidity, the typical attributes of tropical or subtropical climates, have been reported to increase the incidence of blossom-end rot [1], fruit cracking [2], and impaired fruit quality and yield [3, 4]. In recent years, cover cropping techniques such as cultivation within polyethylene rain shelters which provide protection from heavy rain and reduce disease pressure and running cost, resulting in improved crop yield and productivity, have been widely used in the production of fruit trees and other crops.

Tomato (*Solanum lycopersicum* L., syn.* Lycopersicon esculentum* Mill.) is one of the most popular vegetables and an important source of antioxidants including, lycopene, phenolics, and vitamin C (V_C_) in human diet [5]. The yield and quality of fresh tomato are governed by both genetic factors and environmental conditions [6]. Among the environmental factors, water is a major component affecting plant growth and in turn fruit quality of tomatoes. Therefore, optimum irrigation and proper drainage are critical to increase tomato yield and quality. Soil water has been reported to influence crops to develop better roots, enhance nutrient uptake, and contain more leaf area and dry matter [3]. In southern China, soils under rain shelters may experience fluctuating soil water regimes ranging from drought to waterlogging. Tomato would be under strong selective pressure to morphologically adapt to the adverse conditions of periodic waterlogging and drought. Waterlogging affects crop yield [7] through a reduction in shoot development and growth, resulting in reduced amounts of aboveground dry matter. The influence of waterlogging in the reduction of aerial biomass is secondary because it is essentially due to the damage to the root system [8, 9]. Under waterlogging, the root system is directly exposed to the changes in the soil environment including a reduction in the oxygen level and increase in CO_2_ and ethylene concentrations [10]. Under drought conditions, on the other hand, plant roots acclimate to soil water deficiency by developing deep and extensive root systems for increased water extractions [11]. The acclimation to drought is assumed to affect shoot growth and biomass production resulting in poor fruit quality. To our knowledge, there have been fewer experiments studying the effect of rain shelters combined with irrigation and drainage management strategies on growth, fruit yield, and quality characteristics. For tomatoes grown under rain shelters, the growth periods and microclimates were significantly different from open-field and greenhouse conditions. Therefore, it is necessary to investigate the growth, yield, and quality response of tomatoes to different irrigation and drainage treatments under rain-shelter cultivation. We conducted a pilot study to determine the effects of irrigation and drainage management strategies on tomato growth, yield, and fruit quality attributes in the subtropical climate of China.

As the market for fresh vegetable is growing steadily, the need for higher quality produce is increasing [12]. It is an important goal to improve the quality and increase yield simultaneously, but in general the quality improvement is often accompanied by the decline in production [13–16]. Therefore, determination of a suitable irrigation and drainage solution for rain-shelter cultivation with the compromise of water saving, better quality, and higher yield has become an imperative in the production of rain-shelter crops in southern China. Since the principal component and variable fuzzy analysis are simple and the result is easy to understand, they have widely been used to solve many complicated decision making processes in previous studies [17–20]. However, they have not yet been applied in evaluating irrigation and drainage treatments of tomato under rain-shelter cultivation. It is reported that soil water can not synchronously influence fruit yield and quality of tomato under rain shelters [4]. However, there has been little work done to assess the influence of different irrigation and drainage strategies on growth, fruit quality, and yield as an integrated approach. The knowledge on the effects of drainage in tomato is inadequate compared to that of water shortage. We hypothesized that alterations in growing conditions can affect tomato growth from multiple trophic levels, which could have further repercussions on crop productivity. It was also hypothesized that a minimal desiccation applied to can improve fruit quality and delay final harvest. Therefore, the purpose of this paper was to study the impact of different irrigation and drainage strategies on crop growth, fruit quality, and yield in tomatoes. A second objective was to determine the relative membership and relative membership function of the sample indicators of each irrigation and drainage treatment under rain-shelter cultivation relative to the indicators at all levels of the standard range. This could help in calculating the rating scale of each irrigation and drainage treatment by changing the model and its parameters. Ultimately, by selecting the best irrigation and drainage scheme we achieve the goals of a compromise among water saving, better quality, and higher yield.

## 2. Materials and Methods

### 2.1. Experimental Site and Plant Material

The experiments were conducted at the Key Laboratory of Efficient Irrigation-Drainage and Agricultural Soil-Water Environment in Southern China, Ministry of Education (latitude 31°57′ N, longitude 118°50′ E, 144 m above sea level) during the tomato growing season from March to September in 2011 and 2012. The location receives abundant sunshine with mean temperature of 15.7°C, mean sunshine duration of 2017.2 h, and a frost-free period of 224 days. The site lies in a typical subtropical temperate climate zone with pan evaporation of 1472.5 mm and annual precipitation of 1072.9 mm. The experimental field is 18 m long and 7.8 m wide with a planting area of 140 m^2^. The volumetric water content of soil at field capacity and mean dry bulk density was 0.34 cm^3^ *·*cm^−3^ and 1.35 g cm^−3^, respectively, for the upper 30 cm of soil layer. The soil type was clay loam with a pH of 6.1 and the organic matter content was 0.72%. Tomato (var. Xi Lan) seedlings were raised in a nursery and after 5 weeks of sowing they were transplanted at the six-leaf stage. A week before transplanting, the experimental site was ploughed and harrowed to a depth of 25 cm. In all treatments, compound fertilizer (15 : 15 : 15) was applied and incorporated into the soil at the rate of 1200 kg ha^−1^. All the plants were irrigated and allowed to drain to field capacity. After 24 h, the seedlings were transplanted into 15 experimental plots. Each plot consisted of three rows of 2 m in length with spacing of 50 cm between plants and 40 cm between rows. The row at the center was the only one harvested for measurements on crop yield. Immediately after transplanting a light irrigation was provided to ensure seedling establishment. The treatments were imposed two weeks after transplanting. Calcium ammonium nitrate (26% N) fertilizer was applied as side dressing at the rate of 250 kg*·*ha^−1^ in two equal split doses at 5th and 7th weeks after transplanting when the plants were at flowering and first fruit-set stages, respectively. The plots were weeded manually three times during each season. The plants were sprayed with insecticide at the rate of 0.8 l ha^−1^ at the 6th week against fruit worms and other pests.

### 2.2. Treatments and Experimental Design

Under rain shelters, five treatments (T1, T2, T3, T4, and T5; T1 as the control or CK) replicated three times were applied to the experimental units (Table 1). Seven days after transplanting tomatoes were drip-irrigated and the amount of water delivered was recorded using a water gauge. The plants were irrigated to field capacity once average soil volumetric water content in the upper 60 cm layer of the control (CK with no drainage pipe) decreased to 80% of field capacity. Ten days before the final harvest watering was ceased in all the treatments.

**Figure 1:**
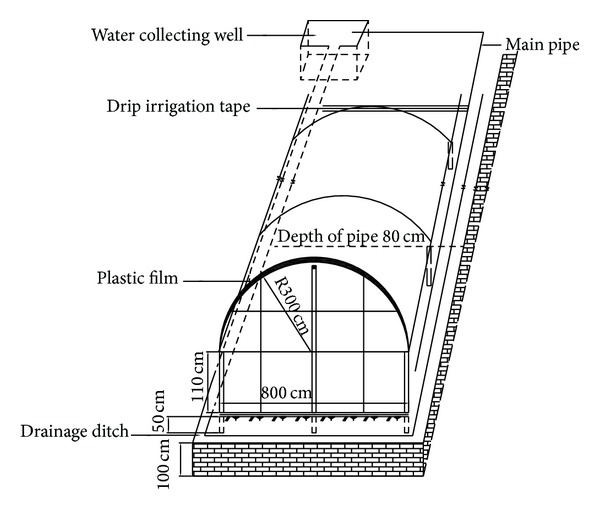
Sketch of the experimental plot under rain shelter.

Rain shelters consisted of polyethylene covers built to prevent rainwater from reaching the tomato canopy. Cross-beams were attached at a height of 1.5 m on top of pillars. Steel posts located every 6 m within the rows to support the trellis system acted as pillars for the frame of the rain shelters. Steel arches (1.5 m span, 0.8 m amplitude) were attached with wire at both ends of the cross-beams. A strip (18 m long, 8 m wide) of greenhouse grade UV-protected transparent polyethylene cover of 0.15 mm thick was stretched over the arches and tied down at the extremities to soil anchors. Bungee cords were snaked through eye bolts and crisscrossed over the rain shelters to fasten the polyethylene strips (Figure 1).

### 2.3. Soil Samples and Water Measurements

Soil samples from all the fifteen plots were collected for the measurement of soil physical and chemical index prior to planting. Soil water contents were used to adjust the irrigation schedule to ensure that the envisaged irrigation treatments were achieved. The total amount of irrigation water was recorded using water meters and drainage volume of groundwater was calculated by water meters connected to the catchment tank. Three perforated PVC pipes (60 mm diameter) were installed to a depth of 150 cm over pipes in three drainage areas and a ruler was inserted into the PVC pipes to measure the depth of the water table every five days. Measurements on soil water content were performed by gravimetric method.

### 2.4. Measurements of Growth

Plant growth characteristics from all the five treatments were determined by six indicators (plant height, root length, root weight, shoot weight, total dry weight, and root-shoot ratio). During final harvest plants were extracted from the soil, washed with distilled water, and divided into three parts: root, stem, and leaf. Roots were stored in FAA (formalin : acetic acid : 70% ethanol = 1 : 1 : 18 v/v) solution for further measurements. Plant height and the length of the primary root were measured using a ruler. Dry weight of all plant fractions was recorded after drying in an oven at 80°C for 48 h. The root-shoot ratio was calculated on dry matter basis (root weight/shoot weight).

### 2.5. Measurements of Yield and Quality

Individual fruit weight and fresh yield of tomato were recorded at each harvesting process. In order to avoid border effects, only the five plants in the row at the center of each plot were used for yield and subsequent quality measurements.

Ripened fruits from the five treatments were sampled at harvest for assessing fruit quality attributes. The tomatoes were washed with running tap water to remove dirt, dried thoroughly with absorbent paper, and then analyzed for mean single fruit weight (MSW) and mean single fruit volume (MSV). V_C_ (ascorbic acid content) was determined by titration of homogenate tomato samples (diluted in a 3% meta-phosphoric acid solution and an 8% acetic acid solution) using a 2,6-dichlorophenol-indophenol solution standardized in a solution of ascorbic acid with a known concentration. Organic acid (OA) was titrated with 0.1 mol*·*L^−1^ NaOH and calculated as equivalents of citric acid expressed as percentage of fresh mass [21]. Total soluble solids (TSS) of tomato juice were measured with a digital refractometer (ATC-1E, ATAGO Master, Japan) at 20°C. In order to avoid cross contamination the refractometer was washed with distilled water and dried with blotting paper after each measurement. Soluble sugar (SS) content was measured using anthrone method [22].

### 2.6. Statistical Analysis

Statistical analyses were performed using SPSS software Version 13.0. Analysis of variance (ANOVA) was performed using the GLM procedure and multiple comparisons of mean values were performed using least significant difference (LSD) test at *P* = 0.05 level. The matrix calculation was done with Matlab 7.0.4 (the Math works, Inc.).

## 3. Determination of Optimal Irrigation and Drainage Treatments with the Compromise of Yield, Quality, and Water Use Efficiency

### 3.1. Principal Component Analysis of Tomato Quality

Assume that the quality index matrix is *A*:

(1) $$\begin{array}{c} A=\left(\begin{array}{ccc} a_{11} & \cdots & a_{1p}\\ \vdots & \ddots & \vdots \\ a_{n1} & \cdots & a_{np} \end{array}\right), \end{array}$$

where *n* is the number of treatments and *p* is the number of quality indicators.

(1) Change the indexes into the same chemotaxis. Use the following formula to change the inverse index:
  (2) $$\begin{array}{c} a_{ij}^{\prime }=-a_{ij}. \end{array}$$
(2) Eliminate the magnitude of every index:
  (3) $$\begin{array}{c} z_{ij}=\frac{\left(a_{ij}^{\prime }-{\bar{a}}_{j}^{\prime }\right)}{s_{j}^{\prime }}, \end{array}$$
  where *z*_*ij*_ is obtained after the standardization of *a*_*ij*_′:
  (4) xj′=∑i=1nxijn,  sj′=∑i=1n(xij′−x−j′)2(n−1)i=1,2,…,n, j=1,2,…,p.
(3) Calculate the correlation matrix of *Z*_*j*_ (*Z*_*j*_ = (*Z*_1*j*_,*Z*_2*j*_,…,*Z*_*nj*_)^*T*^) as follows:
  (5) $$\begin{array}{c} R=\left(\begin{array}{ccc} r_{11} & \cdots & r_{1p}\\ \vdots & \ddots & \vdots \\ r_{p1} & \cdots & r_{pp} \end{array}\right), \end{array}$$
  where *r*_*jk*_ is the correlation coefficient between *Z*_*j*_ and *Z*_*k*_, *k* = 1,2,…, *p*.
(4) Calculate the *k*th principal component *f*_*ik*_ according to *λ*_*k*_ and *α*_*k*_ = (*α*_*k*1_,*α*_*k*2_,…,*α*_*kp*_)^*T*^, where *λ*_*k*_ is the characteristic value of *R* and *α*_*k*_  is the eigenvector. Consider
  (6) $$\begin{array}{c} f_{ik}=\alpha_{k1}z_{i1}+\alpha_{k2}z_{i2}+\cdots \alpha_{kp}z_{ip}, \end{array}$$
  where *z*_*ip*_  (*i* = 1,2,…, *n*) is the evaluation index after standardization processing. Select the *m*  (*m* < *p*) principal components whose cumulative variance contribution rate ∑_*k*=1_^*m*^(*λ*_*k*_/*p*) ≥ 85% as representatives to evaluate.
(5) Use the formula *η*_*k*_ = *λ*_*k*_/*p* to calculate the weight, and then obtain the comprehensive principal components from the following formula:
  (7) $$\begin{array}{c} F_{i}=\eta_{1}f_{i1}+\eta_{2}f_{i2}+\cdots \eta_{m}f_{im}, \end{array}$$
  where *F*_*i*_ is nondimensional. We can convert the *F*_*i*_ to positive by the following formula:
  (8) $$\begin{array}{c} F_{i}^{\prime }=F_{i}+1-\min\left(F_{i}\right). \end{array}$$

### 3.2. Evaluation on Irrigation and Drainage Treatments Based on Variable Fuzzy

It is hypothesized that there are *n* kinds of irrigation and drainage treatments, and these programs form the collection as follows:

(9) $$\begin{array}{c} X=\left\{x_{1},x_{2},\ldots ,x_{n}\right\}. \end{array}$$

Determine the *j*th sample by *m* indicators, and get the eigenvalue matrix:

(10) $$\begin{array}{c} x_{j}=\left(x_{1j},x_{2j},\ldots ,x_{mj}\right). \end{array}$$

Then we can use an *m* × *n* matrix to represent the sample collection

(11) $$\begin{array}{c} X=\left(x_{ij}\right), \end{array}$$

where *x*_*ij*_ is the *i*th index of sample *j*,  *i* = 1,2,…, *m*, *j* = 1,2,…, *n*.

Divide every indicator (the total is *m*) into *c* levels and then obtain a matrix that contains *m* × *c* index values

(12) $$\begin{array}{c} Y=\left(y_{ih}\right), \end{array}$$

where *y*_*ih*_ is the eigenvalues of *H*-level of index *I*, *h* = 1,2,…, *c*.

Determine the attracting (mainly) domain and the range of the matrix domains of irrigation and drainage schemes based on matrix *Y* and the measured data of target irrigation:

(13) $$\begin{array}{c} I_{ab}=\left(\left[a_{ih},b_{ih}\right]\right),\\ I_{cd}=\left(\left[c_{ih},d_{ih}\right]\right). \end{array}$$

Determine the matrix composed of point *M*_*ih*_ (in the attracting (mainly) domain[*a*_*ih*_, *b*_*ih*_]) whose $D_{\underset{\sim }{A}}{\left(x_{ij}\right)}_{h}$ is 1, based on different combinations of level *h* and index *i*:

(14) $$\begin{array}{c} M=\left(M_{ih}\right). \end{array}$$

Determine the positional relationship between the points *x*_*ij*_ and *M*_*ih*_, according to formula from (7) to (9) and then select the formulas as in the work of Chen, 2002 legitimately; then obtain the matrix in which the degree relative differences $D_{\underset{\sim }{A}}{\left(x_{ij}\right)}_{h}$ are 1 and level corresponding indicators about $h\;\mu_{\underset{\sim }{A}}{\left(x_{ij}\right)}_{h}$ are also 1. Consider

(15) $$\begin{array}{c} \left[U_{h}\right]=\left(\mu_{\underset{\sim }{A}}{\left(x_{ij}\right)}_{h}\right). \end{array}$$

Using the model given in [23],

(16) uh′j=1×(1+{∑i=1m[ωi(1−μA~(xij)h)]p∑i=1m(ωiμA~(xij)h)p}α/p)−1,

where _*j*_ *u*_*h*_′ is the relative membership degree before normalization processing, *α* is the optimization criteria of model parameter, *ω*_*i*_ is the weights of index *i*, *m* is the number of index, and *p* is distance parameter (usually take *p* = 1 or *p* = 2).

Calculate the relative membership degree matrix without normalization processing:

(17) U′=(uh′j).

Get the matrix after normalization processing of formula (12):

(18) U=(uhj).

Maximum membership degree principle can not be applied to the evaluation level; characteristic values are used to evaluate the level of the sample [24]. Consider

(19) $$\begin{array}{c} H=\left(1,2,\ldots ,c\right)\times U. \end{array}$$

## 4. Results

### 4.1. Shoot and Root Growth under Different Irrigation and Drainage Treatments

The response of shoot mass to different irrigation and drainage treatments markedly differed in both years (Table 2). Compared to the control plants (CK), T2 and T4 did not significantly affect shoot dry matter production in 2011. However, there was significant difference between them in 2012 (*P* < 0.05). Under drought conditions, T5 had a tendency to decrease shoot mass in both seasons compared with T4, showing significant reductions (17–21%). Furthermore, T3 presented similar trends to T2 with no significant difference at the end of the experiment in both years, compared to the control.

**Figure 2:**
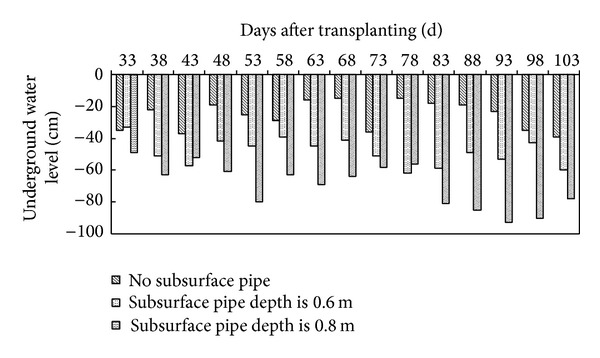
Change of underground water level under three kinds of underground pipe depths.

Root morphological characteristics play an important role in mineral nutrient uptake from the soil [25]. With respect to root production, T2 and T4 had decreased mean root length (5% and 9%, resp.), root mass (15% and 17%, resp.), and root-shoot ratio (6% and 18%, resp.), in both years compared to the CK (Table 2). This difference might be explained by the occasional waterlogging under CK during the growing seasons in 2011 and 2012 (Figure 2). In the case of T3 with 60 cm buried underground pipe, drought stress increased root length by 15%, root mass by 14%, and root-shoot ratio by 39% in both years. As shown in Table 2, compared to T4, a similar trend was observed for T5 in increased root length, root mass, and root-shoot ratio. In general, plants under T3 and T5 had a sharp increase in root length, root mass, and root-shoot ratio and the difference was significant compared with the control (*P* < 0.05).

Considering the treatment effects on plant height and total dry mass, the plants under treatment T2 were the tallest; the differences were significant (*P* < 0.05) in both years compared to the CK (Table 2). The inability of the roots to acclimate to occasional waterlogging under CK might have resulted in reduced growth and function and in turn less dry matter accumulation. In 2012 the total dry mass of the plants in the T2 and T4 increased significantly (*P* ≤ 0.05); however, no significant difference was observed in 2011. Although 13% and 10% total dry mass reduction was evident in the T3 plants in both years, respectively, it was not statistically different from that of T2. On the other hand, compared to T4, drought treatments (T5) exhibited significant (*P* ≤ 0.05) reduction in total biomass in both years.

### 4.2. Single Quality Attributes and Comprehensive Quality Index

#### 4.2.1. Single Quality Attribute

**Figure 3:**
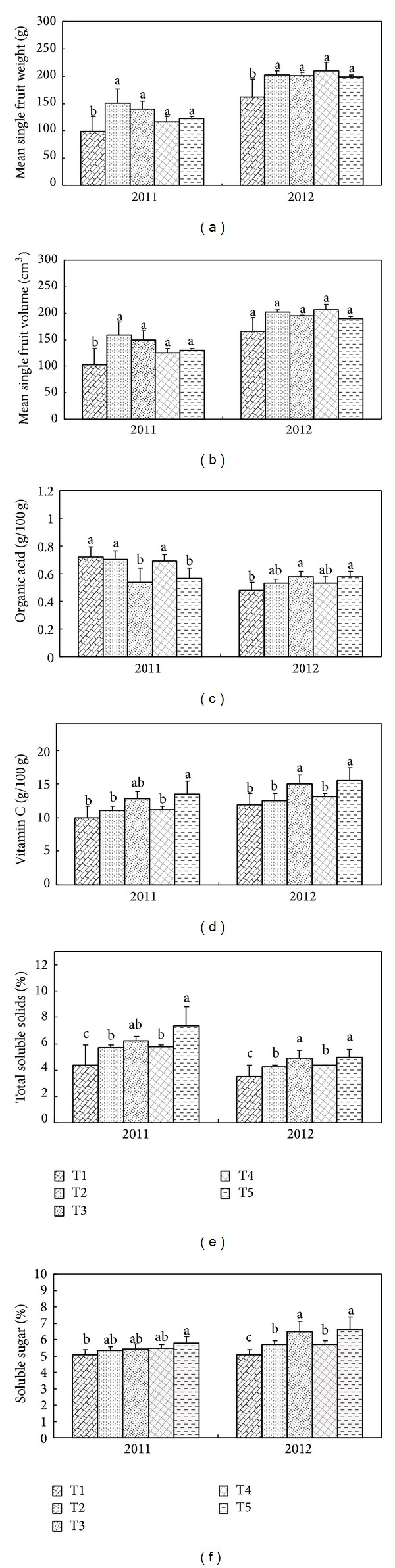
Effects of different irrigation and drainage treatments on quality indices (mean single fruit weight, MSW; mean single fruit volume, MSV; organic acid, OA; vitamin C, V_C_; total soluble solids, TSS; and soluble sugar, SS) of tomato fruit in the 2011 and 2012 seasons. Columns with the same letter represent values that are not significantly different at the 0.05 level of probability according to the LSD test. Vertical bars represent ±S.E. of the mean. The S.E. was calculated across three replicates for each year. The treatment symbols of T1, T2,…, T5 are the same as in Table 1.

Fruit appearance is the first quality trait to consumers and determined by fruit size and shape [26]. In both seasons mean single fruit weight, MSW (Figure 3(a)), was significantly affected by T2 and T4, compared to CK in both years (*P* < 0.05), which indicates that drainage treatment has a significant influence on yield per fruit. The mean single fruit volume, MSV of T2 and T4, was significantly higher than that of CK in 2011 (*P* < 0.05). Although the values for T2 and T4 were higher than that of the CK in 2012, they were not statistically significant. For both seasons, there was no significant difference (*P* > 0.05) in the MSW and MSV between T2 and T3 (Figures 3(a) and 3(b)), indicating that MSW and MSV were not affected by drought.

Tomato taste and nutritional quality are largely determined by the contents of total soluble solids (TSS), organic acid (OA), soluble sugar (SS), vitamin C (V_C_), and the ratio between SS and OA [27]. The effects of the irrigation and drainage treatments on the taste and nutritional qualities of tomato are summarized in Figures 3(c)–3(f). Compared to CK, T2 and T4 did not significantly affect OA in the first year, but it was significant (*P* < 0.05) in the second year (Figure 3(c)). V_C_ is one of the indispensable components of nutrition in fruits. The values of V_C_ increased with less irrigation amount (Figure 3(d)). For well-watered treatments (T2 and T4) the mean V_C_ increased by 8% and 10%, respectively, compared to the CK, although there was no significant reduction in both years (*P* > 0.05). When compared to T2, average V_C_ of T3 increased significantly (*P* < 0.05) by 18% in both seasons. While considering T5, V_C_ increased significantly (20%) as compared to T4 (*P* < 0.05). With respect to TSS (Figure 3(e)), there was significant difference between T2 and T4 compared to CK in 2011 and 2012 (*P* > 0.05). In contrast, SS in T2 and T4 did not increase in the first year (*P* < 0.05) compared to CK, whereas it was significant in 2012 (Figure 3(f)). Under drought conditions, T5 showed a tendency to increase SS in the second year compared with T4, but there was no difference in 2011 (*P* < 0.05).

The sugar-acid ratio of T2 and T4 was 7% and 12% higher than those in CK in the 2011 season and 2% and 7% in the 2012 season, respectively. Compared to T4, the sugar-acid ratio of T5 significantly increased by 30% in 2011 (*P* < 0.05); however, there was no difference in 2012.

#### 4.2.2. Comprehensive Quality Index and Its Response to Different Irrigation and Drainage Treatments

In most cases fruit quality is difficult to be defined because of consumer preferences. In this study, the Principal Component Analysis was attempted to determine the quality component. The results were listed in Tables 3–5. TSS, OA, SS, and V_C_  had high correlation among them while contributing to low correlation with MSW and MSV (Table 3). The accumulated contribution rate of the two principal components which contains a large amount of variation information was 89% (>85%), so the evaluation index can be lower from 6 to 2 (Table 4). The accumulated contribution rate variance of f_1_ is 56.851%. The variation information of f_1_ corresponds to SS (X1), V_C_ (X4), MSW (X5), and MSV (X6) while f_2_ contains the same for TSS (X2) and OA (X3) (Table 4).

According to variable fuzzy model, the results of the comprehensive quality index calculation were shown in Table 5. T3 had the highest comprehensive quality indices in 2011 and 2012 (2.87 and 2.98, resp.), whereas T1 had the lowest index of 1 in both years.

### 4.3. Evaluation on Irrigation and Drainage Treatments with the Compromise among Yield, Quality, and Water Use Efficiency

A scientific irrigation and drainage strategy should be a compromise between water saving and time improving the yield and quality of fruits.  Table 6 shows the results of the water consumption by tomato and fruit yield in the 2011 and 2012. The highest and lowest yields of 156.43 t*·*ha^−1^ and 124.29 t*·*ha^−1^, respectively (2011), and 168.39 t*·*ha^−1^ and 127.32 t*·*ha^−1^, respectively (2012), were obtained from plants grown in similar conditions of the T2 and plants exposed to drought conditions of the T5. There was no significant difference between T2 and T4, compared to the control, indicating that the fruit yield was not benefitted from drainage treatments. The fruit yield was higher when plants were supplied with higher amounts of water. The yields from T3 and T5 were significantly decreased (*P* < 0.05) compared to well-watered plants. In both seasons, T3 and T5 significantly decreased crop water consumption (*P* < 0.05), compared to well-watered treatments.

The weight vector of the four indicators was determined according to the variable fuzzy theorem used to define the importance of the indicators and the consistency [28, 29]; then the qualitative ranking scale matrix can be obtained as follows:

(20) F=[0.501110.511000.50.5000.50.5]②①③③.

Use indicator *x*_2_ with sequence *①* to compare with those of *②*, *③*, and *④*, respectively, and determine the importance according to the method [28] as follows.

Compared to *x*_1_, the importance extent of *x*_2_ is between “little” and “slightly.” Compared to *x*_3_ and *x*_4_, the importance of *x*_2_ is “significant”; then determine the weight vector of four indicators before normalization processing as follows:

(21) $$\begin{array}{c} \omega^{\prime }=\left(\begin{array}{c} 0.714,1,0.333,0.333 \end{array}\right)=\left(\omega_{i}^{\prime }\right). \end{array}$$

The weight vector after normalization processing is as follows:

(22) $$\begin{array}{c} \omega =\left(\begin{array}{c} 0.300,0.420,0.140,0.140 \end{array}\right)=\left(\omega_{i}\right). \end{array}$$

Using the weights provided from this paper and transforming the parameters (*α*, *p*) of the variable fuzzy model, the results of 2011 and 2012 were listed in Table 6. Therefore, considering the water-saving effect, yield, and comprehensive quality, T3 appeared to be the most suitable irrigation and drainage strategy.

## 5. Discussion and Conclusion

Higher yield and better quality in the production of tomatoes stimulated the development of advanced production systems. The production of tomatoes is mostly limited to the hot and wet summer season in southern China. Rain shelters cultivation which can increase crop yield and improve fruit quality, has, therefore, been introduced to this region. The reasonable use of water under rain shelters has become a priority and the adoption of irrigation and drainage strategies that allows saving of irrigation water, maintaining satisfactory yields, and improving fruit quality contributes to the preservation of this option. The effects of irrigation and drainage strategies have been widely investigated for many crops, although these are crop-specific. Moreover, the soil type, which determines the available water for crop uptake, and the growing condition of a given productive cropping system, which determines the growth and water consumption by the crop, play vital roles in determining the effects of irrigation and drainage strategies. Therefore, it is essential to evaluate the impact of different irrigation and drainage strategies with multiyears rain shelter experiments, before suggesting the most appropriate irrigation and drainage strategies to be adopted in southern China for tomatoes.

In southern China, groundwater is usually close to the soil surface for a prolonged period of time due to excessive rainfall during the growing season, which results in a significant risk of intermittent waterlogging (Figure 2). The decline of both plant growth and accumulation and redistribution of dry matter is common among plants sensitive to waterlogging [30]. The roots are the most severely affected plant organ in tomatoes. The decreased availability of both oxygen and photosynthates led to the cessation of growth and progressive deterioration of the main roots. Waterlogging reduces root permeability and hydraulic conductivity, which in turn influences water uptake and transport [31]. These results have been confirmed in this study, where root mass (RM) was significantly reduced under CK without proper drainage. The increased vegetative and reproductive biomass production with drainage treatments might be a possible outcome of the increase in available water and nutrition and as a result of enhanced photosynthetic activity. Treatments T2 and T4 effectively maintained higher SM and TDM than those in the CK treatment. Tomatoes under different buried groundwater pipe depths responded differently to drought, as they did to waterlogging. Several studies have reported a reduction in shoot growth under drought [32]; however, drought treatments can increase the root-shoot ratio. It is widely accepted that the absorption and fixed capacity of the nutrients can be measured by the root dry matter. The root dry matter increases when the soil moisture content is low; in contrast the aboveground dry matter under drought conditions decreases. The larger the root-shoot ratio is, the more water and nutrients can be absorbed by plants, which shows the environmental adaptability of the plant. In the current study, although the treatments T3 and T5 accumulated less amounts of dry matter, the plants adapted to the environment with low water resources by adjusting the distribution ratio of the dry matter and optimizing the plant structure.

Data from this study are comparable to previously published reports on fruit yield under different irrigation and drainage treatments. Under greenhouse conditions, several water-saving systems including deficit irrigation and partial root zone drying have been recommended. These water-saving systems have great potential to conserve water and at the same time improve fruit quality but with yield penalty to various extents. According to Johnstone et al. [33], the total yield increases in relation to the amount of water applied. Other researchers demonstrate that maximum tomato yields are obtainable under irrigation with water amounts based on 100% ETc [34]. In one experiment [35] fruit yield increased when drip irrigation was augmented. In the present study, the yield of T3 and T5 was significantly decreased (*P* < 0.05) compared to well-watered plants. In addition, drainage treatments (T2 and T4) provided the best results by increasing the marketable yield by 13% and 8%, respectively, in two seasons compared with the control. Our findings were in agreement with the results of Comeau et al. [36].

Tomatoes are considered as an important commercial and dietary vegetable. Visual appearance is a critical factor driving the initial consumer's choice. The present study showed that drainage treatments significantly affect MSW and MSV. As expected, fruit size was lower in plants where the least irrigation was applied to in both years. Similar effects of water stress upon fruit weight had been reported previously [37, 38]. MSW was affected by deficit irrigation in applying 60% of full irrigation levels (T2 and T4) throughout the experiment which decreased the MSW. Similar findings were reported previously where MSW and MSV were affected by irrigation water [14, 39].

In addition to the effects on visual appearance, drainage and irrigation treatments are known to influence the taste and nutritional quality of fruits. In the present study, the TSS and SS of freshly harvested fruits were higher with drainage treatments, but no significant difference in the V_C_ of freshly harvested fruits was found (Figure 3(d)). However, the decreased level of irrigation exerted beneficial effects upon fruit quality, mostly with respect to TSS and SS contents. Along with TSS and SS contents, a rise in the V_C_ content of the fruits under drought regimes has been observed which is beneficial for human health. Since V_C_ plays an important role as an antioxidant and protects the plant during oxidative damage by scavenging free radicals and reactive oxygen species (ROS) that are generated by various stresses [40, 41], higher content of V_C_ might maintain relatively lower levels of ROS in tomato fruit, resulting in less damage caused by ROS after drought. Some studies reported that the larger the fruit size, the lower the V_C_ content of tomatoes [37, 42]. Other studies highlighted how V_C_ is positively related to water limitation in the processing of tomatoes [37], although the extent of this effect may also be cultivar-dependent [42]. A similar trend for fruit acidity in response to limited water supply was reported by Wang et al. [43].

Tomato quality is a comprehensive concept and a sum of the interactions among different single quality attributes. It not only includes the external and taste qualities but also involves nutritional and storage qualities [6, 26, 44]. The Principal Component Analysis in the current experiment was attempted to determine the comprehensive quality indices of tomatoes which showed that T3 had the highest comprehensive quality performance during the two growing seasons. The significant positive correlation coefficient between two seasons shows that the ranks of comprehensive quality indices were stable when applying similar irrigation and drainage treatments and therefore reliable.

Multiple attribute decision making is an effective approach to evaluate a set of alternatives, which are characterized in terms of each attribute. The variable fuzzy evaluation model also known as MADM method [45–47] can adopt several weights or change the model (1 linear model and 3 nonlinear models in this paper) by changing parameters (*α* and *p*) on the basis of the primary model. The steps involved in the calculation of the variable fuzzy assessment model and using the same to rank five irrigation and drainage regimes have been detailed elsewhere. The results indicated that T3 can acquire a better compromise among water saving, tomato yield, and fruit quality.

## Figures and Tables

**Table 1:** Treatments of irrigation and drainage of tomato under rain-shelter cultivation.

Treatments	Description
T1 (CK)	Irrigation lower limit is 80% of field capacity, no subsurface pipe drainage
T2	Irrigation lower limit is 80% of field capacity, the buried underground pipe depth is 0.6 m
T3	Compared to T2: 60% water was applied at the irrigation time of T2, the buried underground pipe depth is 0.6 m
T4	Irrigation lower limit is 80% of field capacity, the buried underground pipe depth is 0.8 m,
T5	Compared to T4: 60% water was applied at the irrigation time of T4, the buried underground pipe depth is 0.8 m

CK is the control treatment.

**Table 2:** Effect of different irrigation and drainage treatments (Tr) on plant height (PH), root length (RL), root mass (RM), shoot mass (SM), root-shoot ratio (RSR) and total dry mass (TDM) of tomato at harvest.

Years	Tr	PH/cm	RL/cm	RM/g*·*plant^−1^	SM/g*·*plant^−1^	RSR/g*·*g^−1^	TDM/g*·*plant^−1^
2011	T1	112.33 ± 8.57^b^	34.83 ± 3.35^b^	8.23 ± 1.41^b^	91.60 ± 6.68^ab^	0.09 ± 0.01^b^	99.83 ± 4.27^ab^
	T2	132.33 ± 5.61^a^	37.83 ± 7.03^ab^	10.62 ± 0.84^ab^	102.81 ± 11.30^a^	0.10 ± 0.02^b^	113.43 ± 3.046^a^
	T3	113.00 ± 7.67^b^	42.10 ± 2.75^a^	11.96 ± 1.60^a^	86.34 ± 6.22^ab^	0.14 ± 0.04^a^	98.30 ± 8.81^ab^
	T4	121.33 ± 0.33^ab^	38.73 ± 1.51^ab^	10.69 ± 2.55^ab^	98.13 ± 7.10^a^	0.11 ± 0.02^ab^	108.82 ± 10.35^a^
	T5	112.67 ± 1.33^b^	43.33 ± 4.04^a^	12.49 ± 4.17^a^	81.50 ± 6.77^b^	0.15 ± 0.01^a^	93.99 ± 11.16^b^

2012	T1	86.5 ± 3.59^b^	20.89 ± 0.58^b^	6.12 ± 0.23^b^	78.54 ± 3.86^b^	0.08 ± 0.006^b^	84.66 ± 3.88^b^
	T2	108.1 ± 5.81^a^	22.49 ± 2.96^ab^	7.55 ± 0.93^ab^	94.13 ± 4.63^a^	0.08 ± 0.012^b^	101.68 ± 4.41^a^
	T3	91.4 ± 6.64^b^	27.1 ± 4.50^a^	8.72 ± 1.58^a^	82.69 ± 11.78^ab^	0.11 ± 0.003^a^	91.41 ± 13.31^ab^
	T4	100.1 ± 3.12^ab^	23.69 ± 1.87^ab^	7.82 ± 0.39^ab^	91.95 ± 2.82^a^	0.09 ± 0.006^ab^	99.77 ± 2.57^a^
	T5	87.4 ± 3.10^b^	28.95 ± 1.24^a^	8.85 ± 0.87^a^	72.56 ± 2.90^b^	0.12 ± 0.017^a^	81.41 ± 2.11^b^

Note: The values of plant height (PH), root length (RL), root mass (RM), shoot mass (SM), root-shoot ratio (RSR), and total dry mass (TDM) are the means of 3 replications. In the same column and in the same year, means followed by the same letter (a, b) do not differ significantly at the 5% level by LSD. The treatment symbols of T1, T2,…, T5 are the same as in Table 1.

**Table 3:** The correlation matrix of single quality attributes of tomato.

Years		X1	X2	X3	X4	X5	X6
2011	X1	1					
	X2	0.917	1				
	X3	0.981	0.971	1			
	X4	0.906	0.946	0.933	1		
	X5	0.381	0.725	0.706	0.380	1	
	X6	0.413	0.454	0.344	0.413	−0.997	1

2012	X1	1					
	X2	0.983	1				
	X3	0.992	0.995	1			
	X4	0.976	0.941	0.948	1		
	X5	0.598	0.726	0.679	0.465	1	
	X6	0.427	0.574	0.524	0.273	−0.978	1

Note: The letter codes are X1 (soluble sugar, SS), X2 (total soluble solids, TSS), X3 (organic acid, OA), X4 (Vitamin C, V_C_), X5 (mean single fruit weight, MSW), and X6 (mean single fruit volume, MSV).

**Table 4:** Factors and contribution rates of every main ingredient.

Years	PC	X1	X2	X3	X4	X5	X6	CV	CR	ACR
2011	f_1_	0.783	−0.397	−0.652	0.792	0.970	0.956	3.411	56.851	56.851
	f_2_	0.221	0.875	0.084	0.559	−0.108	−0.115	1.926	32.106	88.958

2012	f_1_	0.955	0992	0.980	0.893	0.807	0.672	4.756	79.268	79.268
	f_2_	−0.290	−0.125	−0.183	−0.439	0.589	0.741	1.222	20.373	99.641

Note: The letter codes are PC (principal components, f_1_ and f_2_), CV (characteristic values), CR (contribution rate), ACR (accumulated contribution rate), X1 (soluble sugar, SS), X2 (total soluble solids, TSS), X3 (Organic acid, OA), X4 (Vitamin C, V_C_), X5 (weight per fruit, MSW), and X6 (volume per fruit, MSV).

**Table 5:** Effect of different irrigation and drainage treatments on yield, comprehensive quality index, water consumption, and irrigation amount of tomato under rain shelters.

Years	Tr	Yield (t/ha)	Comprehensive quality index	Water requirement (mm)	Water irrigation (mm)
2011	T1	137.86^ab^	1.00^b^	309.68^a^	243
	T2	156.43^a^	2.68^a^	310.03^a^	241
	T3	126.43^b^	2.87^a^	195.52^b^	145
	T4	150.71^a^	1.96^ab^	288.48^a^	228
	T5	124.29^b^	2.22^a^	181.70^b^	137

2012	T1	149.66^ab^	1.00^b^	303.75^a^	256
	T2	168.39^a^	2.50^a^	306.78^a^	240
	T3	132.56^b^	2.98^a^	192.66^b^	145
	T4	160.64^a^	2.72^a^	286.57^a^	228
	T5	127.32^b^	2.92^a^	182.82^b^	137

Note: columns with the same letter represent values that are not significantly different at the 0.05 level of probability according to the LSD test. Each value is the mean ± SE (*n* = 3). The treatment symbols of T1, T2,…, T5 are the same as in Table 1.

**Table 6:** Evaluation results of ten treatments of irrigation and drainage by changing two parameters of *α*, *p*.

Years	Treatments	Level eigenvalue				Stability range
		*α* = 1, *p* = 1	*α* = 1, *p* = 2	*α* = 2, *p* = 1	*α* = 2, *p* = 2	
2011	T1	4.596	4.511	4.807	4.753	4.511~4.807
	T2	2.95	2.828	2.66	2.561	2.561~2.950
	T3	2.753	2.806	2.401	2.581	2.401~2.806
	T4	3.643	3.571	3.58	3.507	3.507~3.643
	T5	3.283	3.405	3.163	3.412	3.163~3.412

2012	T1	4.596	4.511	4.807	4.753	4.511~4.807
	T2	2.95	2.828	2.66	2.561	2.561~2.950
	T3	2.753	2.806	2.401	2.581	2.401~2.806
	T4	3.643	3.571	3.58	3.507	3.507~3.643
	T5	3.283	3.405	3.163	3.412	3.163~3.412

Note: *α* is the failure criterion parameter of model optimization and *p* is the distance parameter.

## References

[1] Ho LC, White PJ (2005). A cellular hypothesis for the induction of blossom-end rot in tomato fruit. *Annals of Botany*.

[2] Peet M (1992). Fruit cracking in tomato. *HortTechnology*.

[3] Shao GC, Guo RQ, Liu N (2011). Effects of different irrigation patterns on chlorophyll fluorescence parameters of hot pepper in Southern China. *Transactions of the Chinese Society of Agricultural Engineering*.

[4] Shao GC, Lan JJ, Tong DB, Chen C, Zhang C (2013). Influence of irrigation and drainage mode on water requirement content and fruit yield of tomato cultivated in rain-shelter. *Journal of Drainage and Irrigation Machinery Engineering*.

[5] Toor RK, Savage GP, Heeb A (2006). Influence of different types of fertilisers on the major antioxidant components of tomatoes. *Journal of Food Composition and Analysis*.

[6] Viskelis P, Jankauskiene J, Bobinaite R Content of carotenoids and physical properties of tomatoes harvested at different ripening stages.

[7] Solaiman Z, Colmer TD, Loss SP, Thomson BD, Siddique KHM (2007). Growth responses of cool-season grain legumes to transient waterlogging. *Australian Journal of Agricultural Research*.

[8] Malik AI, Colmer TD, Lambers H, Setter TL, Schortemeyer M (2002). Short-term waterlogging has long-term effects on the growth and physiology of wheat. *New Phytologist*.

[9] Bramley H, Turner DW, Tyerman SD, Turner NC (2007). Water flow in the roots of crop species: the influence of root structure, aquaporin activity, and waterlogging. *Advances in Agronomy*.

[10] Ponnamperuma FN, Kozlowski TT (1984). Effects of flooding on soils. *Flooding and Plant Growth*.

[11] Serraj R, Krishnamurthy L, Kashiwagi J, Kumar J, Chandra S, Crouch JH (2004). Variation in root traits of chickpea (*Cicer arietinum* L.) grown under terminal drought. *Field Crops Research*.

[12] Ruiz-Altisent M, Lleó L, Riquelme F (2006). Instrumental quality assessment of peaches: fusion of optical and mechanical parameters. *Journal of Food Engineering*.

[13] Patanè C, Tringali S, Sortino O (2011). Effects of deficit irrigation on biomass, yield, water productivity and fruit quality of processing tomato under semi-arid Mediterranean climate conditions. *Scientia Horticulturae*.

[14] Patanè C, Cosentino SL (2010). Effects of soil water deficit on yield and quality of processing tomato under a Mediterranean climate. *Agricultural Water Management*.

[15] Wang LJ, Ma G, Zhang LC (2010). Effect of water deficit on fruit quality and production of tomato. *Journal of Anhui Agricultural Science*.

[16] Patanè C, Tringali S, Sortino O (2011). Effects of deficit irrigation on biomass, yield, water productivity and fruit quality of processing tomato under semi-arid Mediterranean climate conditions. *Scientia Horticulturae*.

[17] Al-Harbi KMA (2000). Application of the AHP in project management. *International Journal of Project Management*.

[18] Amiri MP (2010). Project selection for oil-fields development by using the AHP and fuzzy TOPSIS methods. *Expert Systems with Applications*.

[19] Vaidya OS, Kumar S (2006). Analytic hierarchy process: an overview of applications. *European Journal of Operational Research*.

[20] Su YN, Chai CL, Yang YM, Shen P (2007). Variable fuzzy assessment on agricultural eco-environmental quality of Changshu City. *Transactions of the Chinese Society of Agricultural Engineering*.

[21] AOAC Official methods of analysis.

[22] Spiro RG, Neufeld ES, Ginsburg V (1966). Analysis of sugars found in glycoproteins. *Methods in Enzymology. Vol. VIII, Complex Carbohydrates*.

[23] Meng LH, Chen YN, Xu XM, Li WH (2012). Assessing water resources carrying capacity in tarim river basin using a variable fuzzy model. *Bulletin of Soil and Water Conservation*.

[24] Chen SY (2005). Engineering variable fuzzy set theory and model-the fuzzy mathematical foundations of hydrology and water resources. *Dalian University of Technology*.

[25] Sattelmacher B, Gerendas J, Thoms K, Brück H, Bagdady NH (1993). Interaction between root growth and mineral nutrition. *Environmental and Experimental Botany*.

[26] Labate JA, Grandillo S, Fulton T, Munós S, Caicedo A, Peralta I, Kole C (2007). Tomato. *Genome Mapping and Molecular Breeding in Plants: Vegetables*.

[27] Dorais M, Papadopoulos AP, Gosselin A (2001). Greenhouse tomato fruit quality. *Horticultural Reviews*.

[28] Ke LN, Wang QM, Wang GL, Zhang X, Wang FX, Yang J (2013). The design, implementation and application of the seawater quality variable fuzzy evaluation system based on Arc Engine. *Marine Sciences*.

[29] Chen SY (2002). *Fuzzy Recognition Theory and Application of Complex Water Resources System Optimization*.

[30] Chen H, Qualls RG, Miller GC (2002). Adaptive responses of Lepidium latifolium to soil flooding: biomass allocation, adventitious rooting, aerenchyma formation and ethylene production. *Environmental and Experimental Botany*.

[31] Huang B, Wilkinson RE (2000). Waterlogging responses and interaction with temperature, salinity, and nutrients. *Plant Environmental Interaction*.

[32] Dry PR, Loveys BR (1998). Factors influencing grapevine vigour and the potential for control with partial rootzone drying. *Australian Journal of Grape and Wine Research*.

[33] Johnstone PR, Hartz TK, LeStrange M, Nunez JJ, Miyao EM (2005). Managing fruit soluble solids with late-season deficit irrigation in drip-irrigated processing tomato production. *HortScience*.

[34] Bariáin C, Matías IR, Arregui FJ, López-Amo M (2000). Optical fiber humidity sensor based on a tapered fiber coated with agarose gel. *Sensors and Actuators B: Chemical*.

[35] Sanders DC, Howell TA, Hile MMS, Hodges L, Meek D, Phene CJ (1989). Yield and quality of processing tomatoes in response to irrigation rate and schedule. *Journal of the American Society for Horticultural Science*.

[36] Comeau C, Privé J, Moreau G (2012). Beneficial impacts of the combined use of rain shelters and reflective groundcovers in an organic raspberry cropping system. *Agriculture, Ecosystems and Environment*.

[37] Favati F, Lovelli S, Galgano F, Miccolis V, Di Tommaso T, Candido V (2009). Processing tomato quality as affected by irrigation scheduling. *Scientia Horticulturae*.

[38] Ozbahce A, Tari AF (2010). Effects of different emitter space and water stress on yield and quality of processing tomato under semi-arid climate conditions. *Agricultural Water Management*.

[39] Cetin Ö, Uygan D, Boyaci H, Yildirim O Effects of different irrigation treatments on yield and quality of drip-irrigated tomatoes under Eskisehir conditions.

[40] Sanmartin M, Drogoudi PD, Lyons T, Pateraki I, Barnes J, Kanellis AK (2003). Over-expression of ascorbate oxidase in the apoplast of transgenic tobacco results in altered ascorbate and glutathione redox states and increased sensitivity to ozone. *Planta*.

[41] Blokhina O, Virolainen E, Fagerstedt KV (2003). Antioxidants, oxidative damage and oxygen deprivation stress: a review. *Annals of Botany*.

[42] Dumas Y, Dadomo M, Di Lucca G, Grolier P (2003). Effects of environmental factors and agricultural techniques on antioxidant content of tomatoes. *Journal of the Science of Food and Agriculture*.

[43] Wang F, Kang S, Du T, Li F, Qiu R (2011). Determination of comprehensive quality index for tomato and its response to different irrigation treatments. *Agricultural Water Management*.

[44] Marouelli WA, Silva WLC (2007). Water tension thresholds for processing tomatoes under drip irrigation in Central Brazil. *Irrigation Science*.

[45] Li Q, Zhou J, Liu D, Jiang X (2012). Research on flood risk analysis and evaluation method based on variable fuzzy sets and information diffusion. *Safety Science*.

[46] Wang XX, Liang ZM, Huang ZP, Dai CJ (2012). Evaluation of water resources carrying capacity in Wuhan City based on variable fuzzy recognition model. *Water Resources and Power*.

[47] Yan MF, Peng Y, Wang GL (2012). Application of variable fuzzy sets model based on entropy weight in groundwater quality. *Assessment of Dalian*.

